# Metabolomic analysis of skeletal muscle before and after strenuous exercise to fatigue

**DOI:** 10.1038/s41598-021-90834-y

**Published:** 2021-05-27

**Authors:** Hajime Ohmura, Kazutaka Mukai, Yuji Takahashi, Toshiyuki Takahashi

**Affiliations:** Sports Science Division, Equine Research Institute, Japan Racing Association, 1400-4 Shiba, Shimotsuke-shi, Tochigi, 329-0412 Japan

**Keywords:** Biochemistry, Physiology

## Abstract

Thoroughbreds have high maximal oxygen consumption and show hypoxemia and hypercapnia during intense exercise, suggesting that the peripheral environment in skeletal muscle may be severe. Changes in metabolites following extreme alterations in the muscle environment in horses after exercise may provide useful evidence. We compared the muscle metabolites before and after supramaximal exercise to fatigue in horses. Six well-trained horses ran until exhaustion in incremental exercise tests. Biopsy samples were obtained from the *gluteus medius* muscle before and immediately after exercise for capillary electrophoresis–mass spectrometry analysis. In the incremental exercise test, the total running time and speed of the last step were 10.4 ± 1.3 (mean ± standard deviation) min and 12.7 ± 0.5 m/s, respectively. Of 73 metabolites, 18 and 11 were significantly increased and decreased after exercise, respectively. The heat map of the hierarchical cluster analysis of muscle metabolites showed that changes in metabolites were clearly distinguishable before and after exercise. Strenuous exercise increased many metabolites in the glycolytic pathway and the tricarboxylic acid cycle in skeletal muscle. Targeted metabolomic analysis of skeletal muscle may clarify the intramuscular environment caused by exercise and explain the response of working muscles to strenuous exercise that induces hypoxemia and hypercapnia in Thoroughbred horses.

## Introduction

Thoroughbred horses are considered elite athletes because their mass-specific maximal oxygen consumption (*V̇*O_2_max) is approximately two-fold higher than that of elite human athletes^1, 2^. To acquire such high exercise ability, it is thought that Thoroughbred horses need to improve their cardiopulmonary capacity and muscle adaptation. It has been reported that Thoroughbred horses experience severe hypoxemia (partial pressure of oxygen [*P*aO_2_] < 75 mmHg) and hypercapnia (*P*aCO_2_ > 50 mmHg) when exercising supramaximally^1, 3–6^. Therefore, it is understandable that the muscles of the horse are subjected to extreme conditions due to strenuous exercise. Additionally, it has been reported that horses further improve their exercise capacity under hypoxic conditions^2^.

Metabolome analysis, which is the method used for the comprehensive analysis of metabolites, has become common in the field of sports science. However, few studies have performed a metabolome analysis using equine skeletal muscle^7^. Therefore, further studies are warranted to understand muscle adaptations in response to exercise. Currently, there is limited information on muscle metabolism under exercise conditions that induce severe hypoxemia and hypercapnia. We hypothesized that a high-intensity exercise would induce marked changes in the glycolytic pathway, tricarboxylic acid (TCA) cycle and high-energy phosphate compounds in skeletal muscle. Therefore, the purpose of this study was to compare the muscle metabolomics in Thoroughbred horses before and after supramaximal exercise to fatigue.

## Results

### Incremental exercise test

In the incremental exercise test, the total running distance, running time, and speed of the last step were 4,518 ± 939 m, 10.4 ± 1.3 min, and 12.7 ± 0.5 m/s, respectively. Mean *V̇*O_2_max, CO_2_ production, respiratory exchange ratio (RER), heart rate, plasma lactate concentrations, and packed cell volume were 174 ± 10 mL/kg/min, 205 ± 17 mL/kg/min, 1.18 ± 0.07, 222 ± 11 beat/min, 20.5 ± 3.3 mmol/L, and 62.2 ± 3.3%, respectively.

### Metabolome analysis

**Figure 1:**
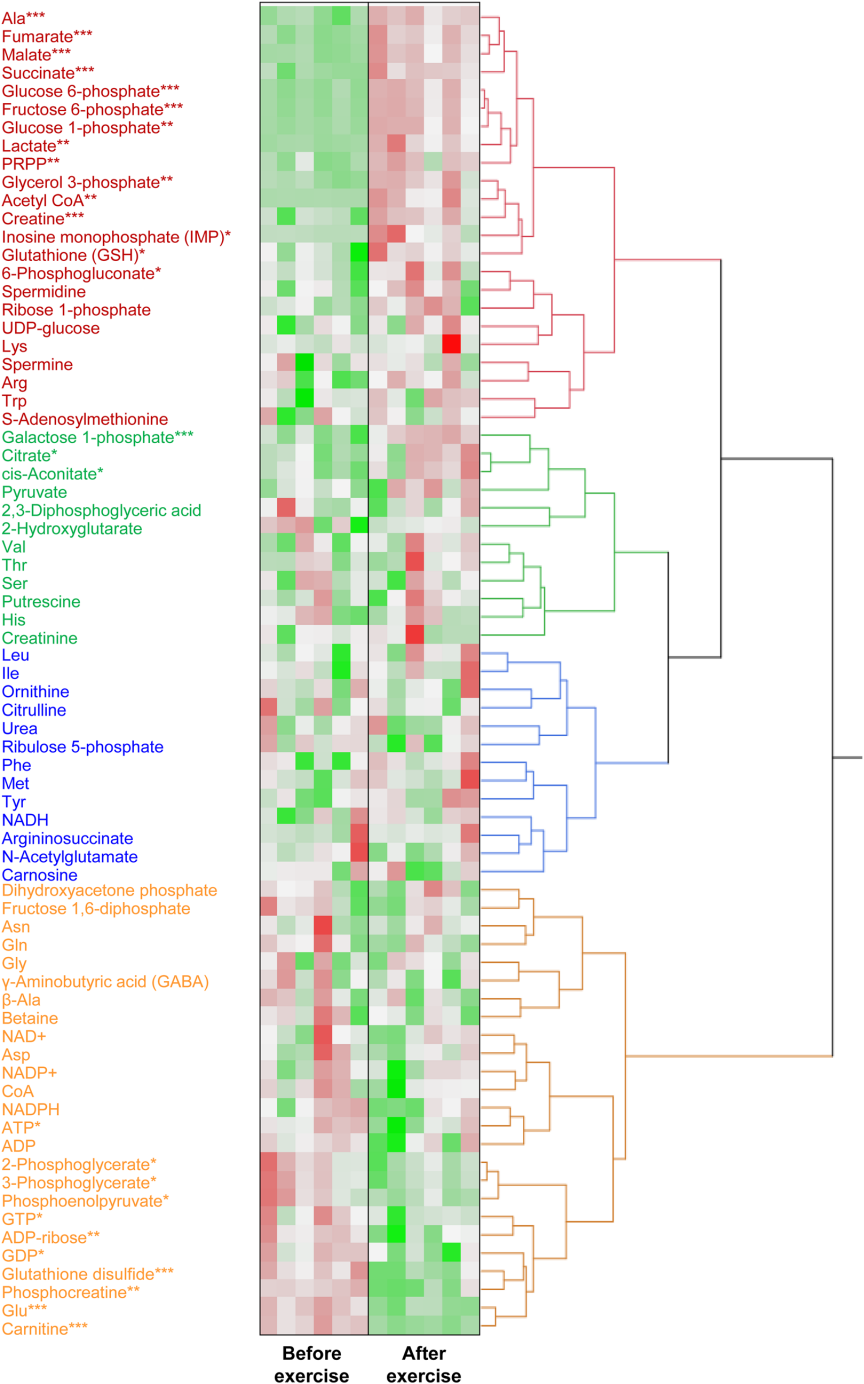
A heat map of the hierarchical cluster analysis of muscle metabolites before and after exercise to fatigue. Each column shows individual horses. Red shows a relatively high concentration of each metabolite. Green shows a relatively low concentration of each metabolite. Asterisks show significant differences between before and after exercise by Welch’s t-test (* < 0.05, ** < 0.01, *** < 0.001).

**Figure 2:**
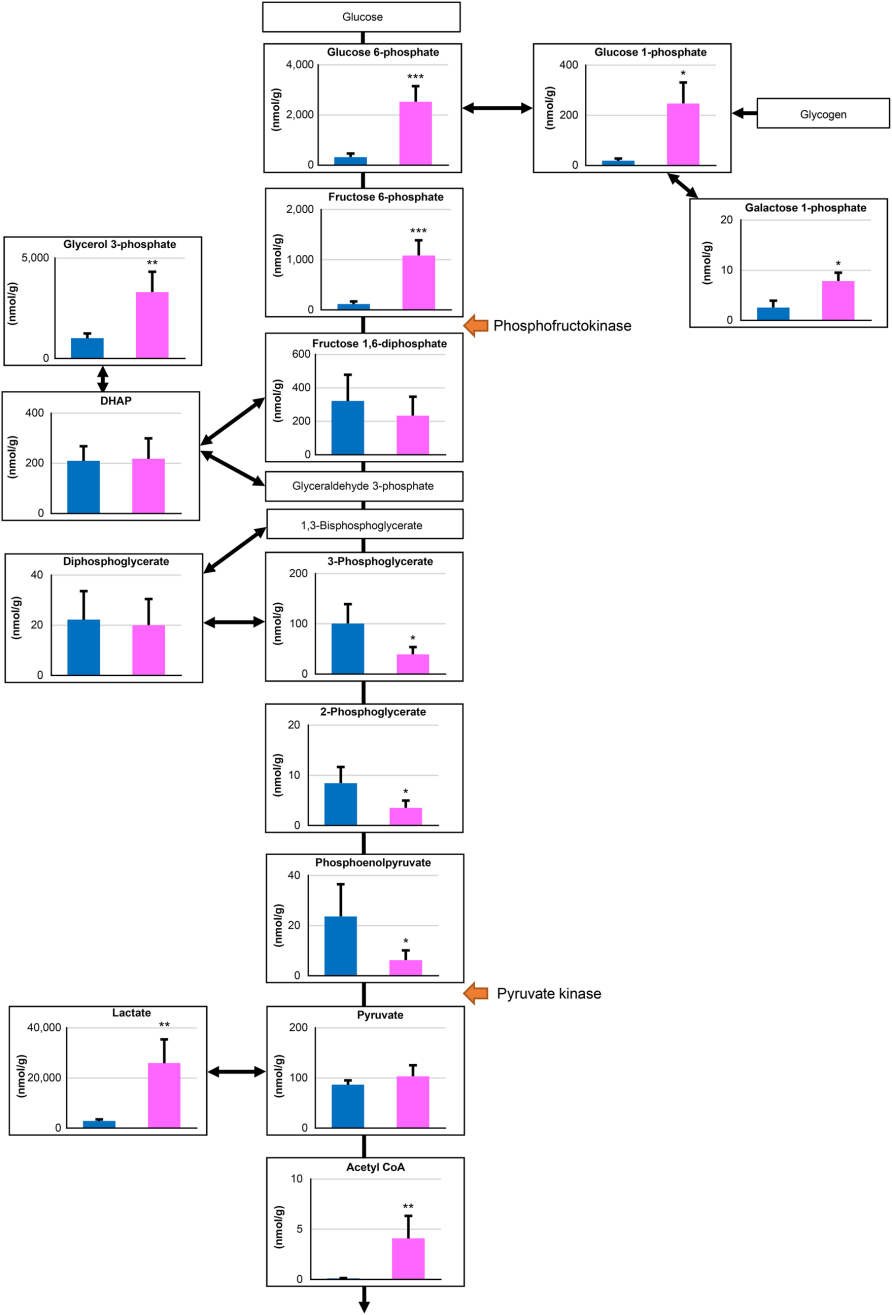
Observed changes in metabolites mapped onto the pathways associated with glycolysis. Asterisks show significant differences between before and after exercise by Welch’s t-test (* < 0.05, ** < 0.01, *** < 0.001).

**Figure 3:**
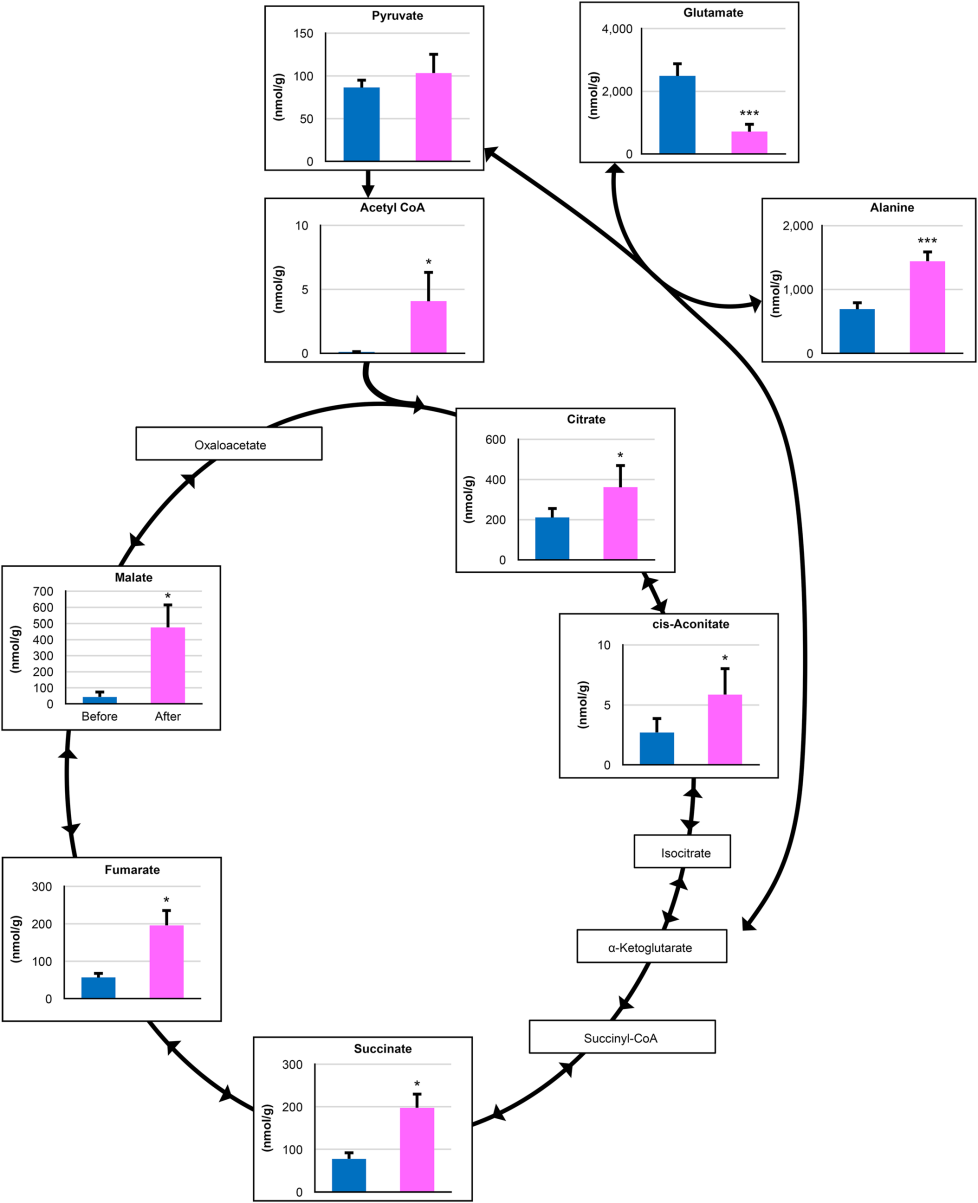
Observed changes in metabolites mapped onto the pathways associated with the TCA cycle. Asterisks show significant differences between before and after exercise by Welch’s t-test (* < 0.05, ** < 0.01, *** < 0.001).

We detected 73 metabolites from the muscle samples (Supplementary Table 1, Fig. 1). Of those, 18 and 11 metabolites were significantly increased and decreased, respectively, after exercise. Many metabolites involved in glycolysis and the TCA cycle, such as glucose 6-phosphate (320 vs. 2,529 nmol/g), acetyl coenzyme A (CoA) (0.1 vs. 4.1 nmol/g), and succinic acid (77 vs. 197 nmol/g), were increased after exercise (Supplementary Table 1, Figs. 1, 2, 3). In contrast, glutamate (2488 vs. 713 nmol/g) and carnitine (5881 vs. 1474 nmol/g) were decreased after exercise (Supplementary Table 1). There were no changes in branched-chain amino acids.

## Discussion

We performed a targeted quantitative metabolomic analysis focusing on energy metabolites to investigate the changes induced by high-intensity exercise in the glycolytic pathway, TCA cycle, pentose phosphate pathway, amino acids, and high-energy phosphate compounds in equine skeletal muscle. The heat map of the hierarchical cluster analysis of muscle metabolites showed that changes in metabolites were clearly distinguishable before and after exercise. Eighteen and 11 metabolites were significantly increased and decreased, respectively, due to strenuous exercise. Numerous metabolites involved in the TCA cycle, some metabolites of glycolysis, lactate, and alanine were significantly increased after exercise and are classified in the same cluster. Some metabolites of glycolysis, phosphocreatine, glutamate, and glutathione disulfide were significantly decreased after exercise and are classified in the same cluster. The changes in several metabolites may have prevented the continuation of strenuous exercise. Based on the intensity and duration of the bout and accumulation of lactate, the exercise performed in this study was considered supramaximal exercise for Thoroughbreds. The observed changes in metabolomics may vary depending on the amount, duration, and intensity of the exercise as well as the test animal. In addition, the small n size is a limitation of this study.

For the production of energy, glycolysis is initiated from glucose 6-phosphate that is phosphorylated glucose. In this study, the levels of glucose 6-phosphate were significantly increased (eight-fold change) after exercise. Similarly, the levels of fructose 6-phosphate were also significantly increased after exercise. Figure 2 shows that the concentrations of these two metabolites were relatively higher than those of other metabolites involved in the glycolytic pathway. In contrast, the levels of metabolites from fructose 1,6-diphosphate to phosphoenolpyruvate involved in the glycolytic pathway decreased after exercise. Fructose 1,6-diphosphate is produced by the catalytic action of phosphofructokinase, which is a key regulator of the glycolytic pathway. Phosphoenolpyruvate is the substrate of pyruvate kinase that irreversibly converts phosphoenolpyruvate to pyruvate. The concentrations of three metabolites (3-phosphoglycerate, 2-phosphoglycerate, and phosphoenolpyruvate) between the enzymes phosphofructokinase and pyruvate kinase were relatively lower than that of the two metabolites glucose-6-phosphate and fructose-6-phosphate. Hence, they may be rapidly converted to the downstream metabolite, and their levels subsequently decrease after the exercise. These results indicate that the exercise affected the flux of the glycolytic pathway, and the changes in metabolites may be different at the point of enzymes that are key regulators or react irreversibly. In addition, although there was no change in pyruvate, the levels of lactate and acetyl CoA (products of pyruvate) were significantly increased after exercise. We believed that the increase in metabolites of the upstream and downstream in glycolysis showed that many substrates have flowed into this pathway. Therefore, it appears reasonable to conclude that the increased levels of glycolysis metabolites may indicate activation of this pathway by exercise.

All metabolites we detected in the TCA cycle were significantly increased by strenuous exercise in this study (Fig. 3). Gibala et al*.* reported that the TCA cycle intermediates were increased during exercise and this increase was primarily mediated by large changes in succinate, malate, and fumarate^8^. We found similar changes in these three metabolites as well as an increase in citrate. Similar to glycolysis, the observed increase in metabolites was attributed to the increased supply of metabolites as a result of the activated TCA cycle by strenuous exercise.

It is well established that several amino acids are converted into intermediates in the TCA cycle. Following activation of the anaplerotic process by strenuous exercise, the concentrations of amino acids involved in this process change. In this study, glutamate was significantly decreased, whereas alanine was significantly increased after strenuous exercise. Pyruvate and glutamate are reversibly catalyzed to alanine and α-ketoglutarate by alanine transaminase. Glutamate is also reversibly catalyzed to α-ketoglutarate by glutamate dehydrogenase or a glutamate-linked aminotransferase^9^. These two pathways appear to be catalyzed toward increasing the levels of α-ketoglutarate by exercise. It has been demonstrated that the alanine aminotransferase reaction is the most important anaplerotic process during the initial minutes of contraction in human skeletal muscle^8^. In addition, studies have shown that α-ketoglutarate was the only TCA cycle intermediate to decrease at the onset of exercise^8, 10^. Therefore, two reactions may supplement α-ketoglutarate during exercise. These reports are consistent with our results, implying that the noted decrease in glutamate and the increase in alanine may be due to the result of anaplerosis of the TCA cycle. However, we did not find any changes in aspartate that potentially participates in the anaplerotic reactions. These results suggest that the changes in alanine and glutamate may be the main indicator of the anaplerotic flux of amino acids into the TCA cycle during exercise.

Branched-chain amino acids (BCAAs) are an important source of substrates for the TCA cycle. Recently, it was reported that the concentrations of BCAAs in blood may be related to central fatigue during exercise^11–13^. In this study, there were no changes in valine, leucine, or isoleucine in skeletal muscle before and after strenuous exercise. It is proposed that BCAAs may not play an important role as an energy source during short duration, high-intensity exercise and may be irrelevant to the fatigue induced by the exercise.

**Figure 4:**
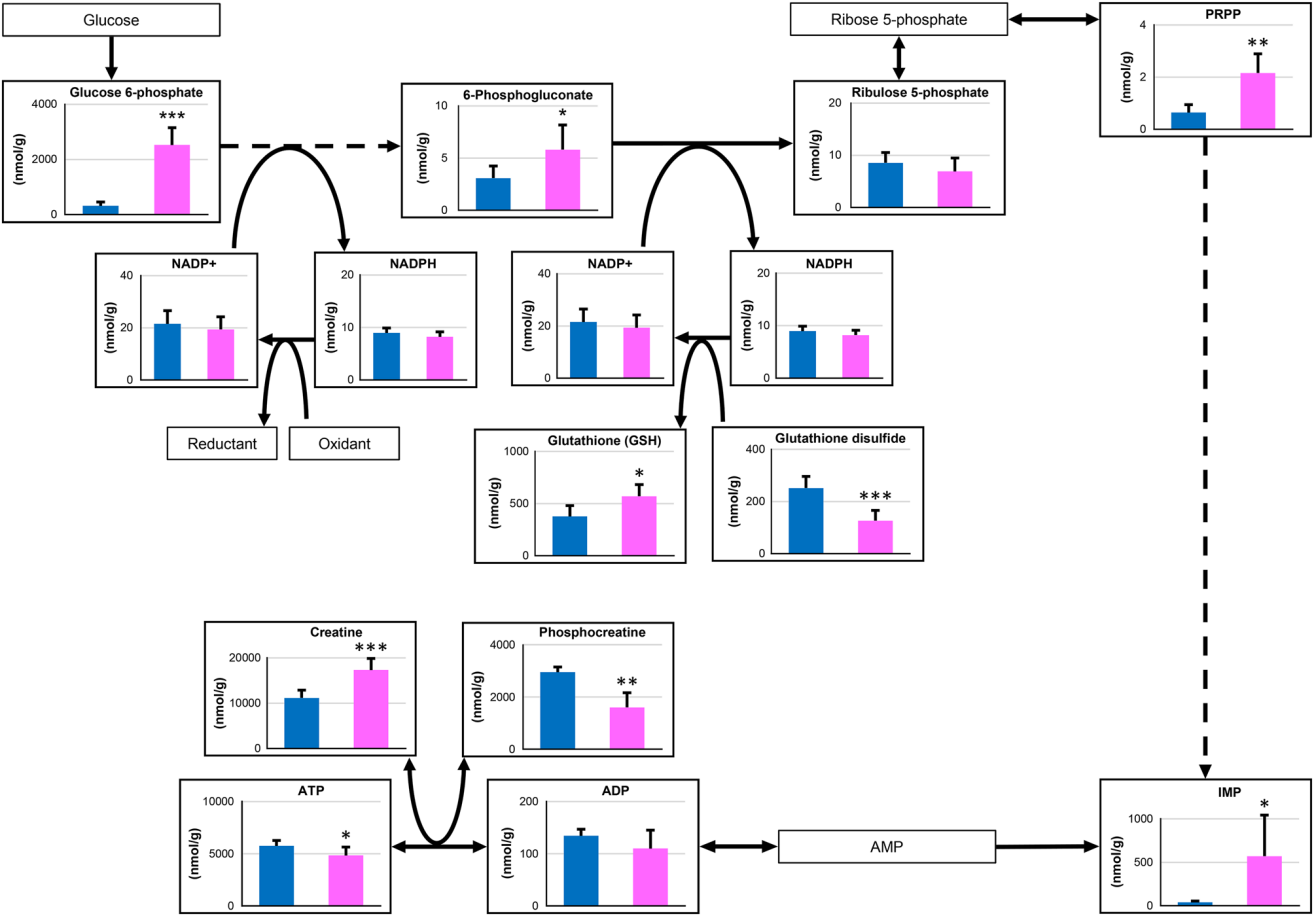
Observed changes in metabolites mapped onto the pathways associated with pentose phosphate pathway and high-energy phosphate compounds. Asterisks show significant differences between before and after exercise by Welch’s t-test (* < 0.05, ** < 0.01, *** < 0.001).

Phosphocreatine is reversibly catalyzed to creatine by creatine kinase and its high-energy phosphate that can be reversibly transferred from adenosine diphosphate (ADP) to adenosine triphosphate (ATP). Guanosine triphosphate (GTP) also contains high-energy phosphate that is reversibly transferred to ATP. In the present study, we observed that these metabolites were significantly decreased after exercise (phosphocreatine: 0.26-fold; ATP: 0.84-fold; GTP: 0.81-fold; guanosine diphosphate: 0.83-fold) (Fig. 4). The decrease in ADP was not significant (0.82-fold); however, ADP and ATP were classified in the same cluster by the hierarchical cluster analysis. The intramuscular concentration of GTP was markedly lower than that of ATP and phosphocreatine, and the change in GTP may not affect the energy requirements of muscle. The exercise performed in this study was supramaximal exercise in Thoroughbreds. Therefore, it is reasonable to observe significant decreases in the levels of phosphocreatine, ATP, and ADP. These results were consistent with the report by McGowan et al.^14^. It is unclear whether the decreased high-energy phosphate in the muscle directly induced exhaustion. Nevertheless, the decrease in phosphocreatine was marked compared with those of the other high-energy phosphates in the muscle. As a result, we observed a significant increase (1.55-fold change) in creatine, a metabolite of phosphocreatine, after the strenuous exercise.

Carnitine plays an important role in transporting long-chain fatty acids from the cytosol into mitochondria; this process is termed the “carnitine shuttle.” Long-chain fatty acids in mitochondria are utilized for energy production via β-oxidation^15^. It has been reported that acetylcarnitine in plasma, a metabolite of carnitine, was increased by submaximal exercise^16^. In contrast, Furuichi et al*.* reported that the concentration of carnitine in muscle did not increase by electric muscle contraction as heavy muscle load, whereas that of acetylcarnitine was significantly increased^17^. In this study, carnitine (0.25-fold change) was one of the metabolites that showed the largest decrease after the strenuous exercise. This may indicate that β-oxidation was also very active even during the strenuous exercise in Thoroughbred horses. From our previous experience, the type of exercise employed in this study results in an RER which exceeds 1.0 at exhaustion^1, 2, 18, 19^. Consistent with these results, the RER in this study was 1.18 ± 0.07. Although an RER > 1.0 indicates that carbohydrate is the predominant source of substrates, a significant reduction in carnitine may indicate that fat is also utilized during strenuous exercise. However, based on the RER, we hypothesized that the contribution of fat to the total energy expenditure may be low in this type of exercise in horses.

The first two steps of the pentose phosphate pathway from glucose 6-phosphate to ribulose 5-phosphate result in the production of two nicotinamide adenine dinucleotide phosphate (NADPH) molecules, which is a major reductant with antioxidant activity. Glucose 6-phosphate dehydrogenase irreversibly converts glucose-6-phosphate to 6-phosphgluconate and is a key regulator of the pentose phosphate pathway. The observed increase in 6-phosphgluconate (1.88-fold change) indicated the activation of the pentose phosphate pathway by the exercise (Fig. 4). Although the levels of NADPH (0.92-fold change) were maintained after exercise, it is known that glucose 6-phosphate dehydrogenase is activated by decreasing NADPH. In this study, the concentration of reduced glutathione (GSH) (1.51-fold change), which is deoxidized by NADPH, was significantly increased. Reportedly, activation of the pentose phosphate pathway may be involved in the resistance against reactive oxygen species and amplified NADPH production^20^. Gohil et al*.* reported that submaximal cycle ergometer exercise for 90 min decreased the levels of GSH, whereas those of oxidized GSH (glutathione disulfide) increased in blood^21^. This observation was inconsistent with our results. However, the differences in exercise intensity and specimens (blood vs. muscle) analyzed may have led to different results.

Phosphoribosyl pyrophosphate (PRPP) is a substrate upstream of inosine monophosphate (IMP). As a result of the activation of the pentose phosphate pathway, the concentrations of PRPP and IMP were significantly increased (3.34- and 14.3-fold change, respectively) by strenuous exercise (Fig. 4). However, IMP is also generated by consuming the high-energy phosphates of ATP and ADP. Therefore, the increase in IMP may be due to both the degradation of ADP and an increase in supply from the pentose phosphate pathway.

The majority of fibers of the gluteal muscle in Thoroughbred horses are type IIa and IIx fibers^22–24^. It has also been reported that the recruitment patterns of muscle fibers differ depending on the intensity and duration of exercise. For example, short duration/high-intensity exercise stimulated type IIx fiber and IIa/IIx fibers^22^. Therefore, these results may reflect the metabolomic changes in only these fast-twitch muscles after exercise in Thoroughbred horses. It is known that different fiber types have different mitochondrial content^22^. The results of the metabolomic analysis may vary depending on the type of muscle fiber and/or pattern of exercise. Further studies are warranted to investigate these hypotheses. The detection of changes in the muscle metabolome may help clarify the alterations caused by exercise in the intramuscular environment and the mechanisms through which working muscles adapt to strenuous exercise that induces hypoxemia and hypercapnia in Thoroughbred horses.

## Methods

### Ethics statement

This study was conducted at the Equine Research Institute, Japan Racing Association (Tochigi, Japan), and its protocol was reviewed and approved by the institutional Animal Use and Care Committee (Approval No.: 19-23, Approval date: 14 June 2019). All experiments were performed in accordance with our institutional guidelines. All authors confirm that the authors complied with the ARRIVE guidelines. Consent to participate/Consent to publish is not applicable.

### Horses

Six healthy Thoroughbreds (three geldings and three females; age: 6.0 ± 1.7 (mean ± SD) years; weight: 495 ± 36 kg) were studied. The horses were trained for 1 month on an equine treadmill by running up a 6% incline twice a week prior to the experiment and veterinarians examined their health every week. The exercise protocol consisted of 2-min exercise intervals at 1.7, 4.0, 7.0, 10.0, 12.0, and 13.0 m/s. On 3 days, the horses walked at a speed of 7 km/h for 1 h in a walking machine; for the other 2 days of the week, the horses rested in their stalls.

### Incremental exercise test

Incremental exercise tests to exhaustion were performed on a treadmill inclined to a 6% grade as a single bout for each horse. Before leading a horse onto the treadmill, a 14-gage Teflon catheter was placed in the left jugular vein following injection of a local anesthetic agent (lidocaine; Fujisawa Pharmaceutical Co., Osaka, Japan) and a heart rate monitor (S810; Polar, Kempele, Finland) was put to measure the heart rate. The exercise protocol consisted of warm-up (1.7 m/s for 2 min and 3.5 m/s for 3 min), followed by 2-min exercise intervals at 1.7, 4.0, 7.0, 10.0, 12.0, and 13.0 m/s until the horse could not maintain its position at the front of the treadmill with humane encouragement. During exercise, a mask was placed on the horse to collect exhaled breath, and oxygen consumption and CO_2_ production were measured as previously described^19, 25^. Biopsy samples for the metabolomic analysis were obtained from the same area (3 cm away from the first sampling point) at the midsection of the *gluteus medius* muscle and from the same depth (5 cm below the skin surface) using a 14-gage needle under local anesthesia (lidocaine; Fujisawa Pharmaceutical Co.) before and immediately after exercise on the treadmill. Muscle samples were rapidly frozen in liquid nitrogen and stored at − 80 °C until the metabolomic analysis. Venous blood was drawn from the jugular catheter to measure the concentration of lactate in plasma and packed cell volume. Blood samples were centrifuged (KH120A, Kubota, Tokyo, Japan) for 5 min (12,000×*g*) to measure the packed cell volume. The plasma was separated through centrifugation for 5 min (1800×*g*) to measure the concentration of lactate using a lactate analyzer (BIOSEN C-LINE Glucose Lactate Analyzer; EKF-diagnostic GmbH, Barleben, Germany). The running distance was calculated as the sum of the running speed × 2 min at each running step.

### Measurement of metabolites

Metabolome measurements were performed by Human Metabolome Technologies, Inc. (Tsuruoka, Japan). Frozen muscle (~ 40 mg) was added to 750 µL of 50% acetonitrile/Milli-Q water containing internal standards (H3304-1002; Human Metabolome Technologies, Inc.) at 0 °C to inactivate enzymes. The muscle was homogenized (thrice at 3500 rpm for 120 s) using a tissue homogenizer (Micro Smash MS100R; Tomy Digital Biology Co., Ltd., Tokyo, Japan), and the homogenate was centrifuged (2300×*g* at 4 °C for 5 min). Subsequently, 400 µL of the upper aqueous layer was centrifugally filtered through a Millipore 5-kDa cutoff filter (9100×*g* at 4 °C for 120 min) to remove proteins. The filtrate was centrifugally concentrated and re-suspended in 50 µL of Milli-Q water for capillary electrophoresis–mass spectrometry (CE-MS) analysis.

According to the methods developed by Soga et al., cationic compounds were measured in the positive mode of CE-time of flight MS (CE-TOFMS), while anionic compounds were measured in the positive and negative modes of CE-MS/MS^26–28^. Peaks detected by CE-TOFMS and CE-MS/MS were extracted using and automatic integration software (MasterHands; Keio University, Tsuruoka, Japan^29^ and MassHunter Quantitative Analysis B.04.00; Agilent Technologies, Santa Clara, CA, USA, respectively) to obtain peak information including *m/z*, migration time (MT), and peak area. The peaks were annotated with putative metabolites from the Human Metabolome Technologies metabolite database based on their MTs in CE and *m/z* values determined by TOFMS and MS/MS. The tolerance range for the peak annotation was configured at ± 0.5 min for MT and ± 10 ppm for *m/z*. In addition, the concentrations of metabolites were calculated by normalizing the peak area of each metabolite with respect to the area of the internal standard and using standard curves, which were obtained by three-point calibrations.

### Statistical analysis

All data are presented as the mean ± standard deviation. Hierarchical cluster analysis using a standardized value was performed by the JMP 13.1 (SAS Institute Inc., Cary, NC, USA) software. Comparisons were conducted using Welch’s t-test (*P* < 0.05).

## Supplementary Information

Supplementary Information.

## Data Availability

The datasets generated during and/or analyzed during the current study are available from the corresponding author on reasonable request.

## References

[1] Ohmura H, Matsui A, Hada T, Jones JH (2013). Physiological responses of young thoroughbred horses to intermittent high-intensity treadmill training. Acta Vet. Scand..

[2] Ohmura H, Mukai K, Takahashi Y, Takahashi T, Jones JH (2017). Hypoxic training increases maximal oxygen consumption in Thoroughbred horses well-trained in normoxia. J. Equine Sci..

[3] Erickson BK (1995). Hypoxic helium breathing does not reduce alveolar-arterial PO2 difference in the horse. Respir. Physiol..

[4] Ohmura H, Hiraga A, Jones JH (2013). Exercise-induced hypoxemia and anaerobic capacity in Thoroughbred horses. J. Phys. Fitness Sports Med..

[5] Wagner PD (1996). Effects of altered FIO2 on maximum VO2 in the horse. Respir. Physiol..

[6] Wagner PD (1989). Mechanism of exercise-induced hypoxemia in horses. J. Appl. Physiol..

[7] Klein DJ, McKeever KH, Mirek ET, Anthony TG (2020). Metabolomic response of equine skeletal muscle to acute fatiguing exercise and training. Front. Physiol..

[8] Gibala MJ, MacLean DA, Graham TE, Saltin B (1997). Anaplerotic processes in human skeletal muscle during brief dynamic exercise. J. Physiol..

[9] Cooper AJL, Kuhara T (2014). α-Ketoglutaramate: An overlooked metabolite of glutamine and a biomarker for hepatic encephalopathy and inborn errors of the urea cycle. Metab. Brain Dis..

[10] Gibala MJ, Tarnopolsky MA, Graham TE (1997). Tricarboxylic acid cycle intermediates in human muscle at rest and during prolonged cycling. Am. J. Physiol..

[11] Chang CK (2015). Branched-chain amino acids and arginine improve performance in two consecutive days of simulated handball games in male and female athletes: A randomized trial. PLoS ONE.

[12] Mikulski T, Dabrowski J, Hilgier W, Ziemba A, Krzeminski K (2015). Effects of supplementation with branched chain amino acids and ornithine aspartate on plasma ammonia and central fatigue during exercise in healthy men. Folia Neuropathol..

[13] Chen IF, Wu HJ, Chen CY, Chou KM, Chang CK (2016). Branched-chain amino acids, arginine, citrulline alleviate central fatigue after 3 simulated matches in taekwondo athletes: a randomized controlled trial. J. Int. Soc. Sports Nutr..

[14] McGowan CM, Golland LC, Evans DL, Hodgson DR, Rose RJ (2002). Effects of prolonged training, overtraining and detraining on skeletal muscle metabolites and enzymes. Equine Vet. J. Suppl..

[15] Bremer J (1983). Carnitine–metabolism and functions. Physiol. Rev..

[16] Davison G (2018). Metabolomic response to acute hypoxic exercise and recovery in adult males. Front. Physiol..

[17] Furuichi Y (1837). Imaging mass spectrometry reveals fiber-specific distribution of acetylcarnitine and contraction-induced carnitine dynamics in rat skeletal muscles. Biochim. Biophys. Acta.

[18] Ohmura H, Hiraga A, Jones JH (2006). Method for quantifying net anaerobic power in exercising horses. Equine Vet. J. Suppl..

[19] Ohmura H, Mukai K, Matsui A, Takahashi T, Jones JH (2020). Cardiopulmonary function during supramaximal exercise in hypoxia, normoxia and hyperoxia in Thoroughbred horses. J. Equine Sci..

[20] Kuehne A (2015). Acute activation of oxidative pentose phosphate pathway as first-line response to oxidative stress in human skin cells. Mol. Cell.

[21] Gohil K, Viguie C, Stanley WC, Brooks GA, Packer L (1988). Blood glutathione oxidation during human exercise. J. Appl. Phys..

[22] Yamano S, Eto D, Hiraga A, Miyata H (2006). Recruitment pattern of muscle fibre type during high intensity exercise (60–100% VO2max) in thoroughbred horses. Res. Vet. Sci..

[23] Kawai M (2009). Muscle fiber population and biochemical properties of whole body muscles in Thoroughbred horses. Anat. Rec. (Hoboken).

[24] Yamano S, Kawai M, Minami Y, Hiraga A, Miyata H (2010). Differences in muscle fiber recruitment patterns between continuous and interval exercises. J. Equine Sci..

[25] Birks EK, Ohmura H, Jones JH (2019). Measuring VO2 in hypoxic and hyperoxic conditions using dynamic gas mixing with a flow-through indirect calorimeter. J. Equine Sci..

[26] Soga T, Heiger DN (2000). Amino acid analysis by capillary electrophoresis electrospray ionization mass spectrometry. Anal. Chem..

[27] Soga T (2002). Simultaneous determination of anionic intermediates for *Bacillus subtilis* metabolic pathways by capillary electrophoresis electrospray ionization mass spectrometry. Anal. Chem..

[28] Soga T (2003). Quantitative metabolome analysis using capillary electrophoresis mass spectrometry. J. Proteome Res..

[29] Sugimoto M, Wong DT, Hirayama A, Soga T, Tomita M (2010). Capillary electrophoresis mass spectrometry-based saliva metabolomics identified oral, breast and pancreatic cancer-specific profiles. Metabolomics.

