# Supplementary material for: Metabolomic analysis of skeletal muscle before and after strenuous exercise to fatigue
Source: Sci Rep. 2021 May 27;11:11261. doi: 10.1038/s41598-021-90834-y (PMC8160181; doi:10.1038/s41598-021-90834-y)
Supplement: Supplementary file 1 — Supplementary Information. [file 41598_2021_90834_MOESM1_ESM.docx]

**Supplementary Table**

**Supplementary Table 1.** **Muscle metabolites before and after exercise.**

| Name | Before exercise | After exercise | Fold change |  | |  |
| --- | --- | --- | --- | --- | --- | --- |
| Acetyl CoA | 0.10 ± 0.03 | 4.08 ± 2.25 | 41.95 ** | | | |
| Inosine monophosphate | 40 ± 16 | 570 ± 473 | 14.29* | | | |
| Glucose 1-phosphate | 18.8 ± 8.7 | 247 ± 84 | 13.16** | |  |  |
| Malate | 43 ± 30 | 477 ± 138 | 11.07*** | |  |  |
| Fructose 6-phosphate | 116 ± 51 | 1,085 ± 301 | 9.36*** | |  |  |
| Lactate | 2,888 ± 660 | 25,917 ± 9,493 | 8.97** | |  |  |
| Glucose 6-phosphate | 320 ± 141 | 2,529 ± 622 | 7.89*** | |  |  |
| Fumarate | 57 ± 11 | 96 ± 39 | 3.45*** | |  |  |
| Phosphoribosyl pyrophosphate | 0.6 ± 0.3 | 2.2 ± 0.71 | 3.34** | |  |  |
| Glycerol 3-phosphate | 1012 ± 233 | 3,310 ± 1,018 | 3.27** | |  |  |
| Galactose 1-phosphate | 2.6 ± 1.4 | 7.9 ± 1.7 | 3.04*** | |  |  |
| Succinate | 77 ± 14 | 197 ± 32 | 2.55*** | |  |  |
| Cis-aconitate | 2.7 ± 1.2 | 5.9 ± 2.2 | 2.16* | |  |  |
| Alanine | 692 ± 100 | 1442 ± 146 | 2.08*** | |  |  |
| Ribose 1-phosphate | 5.0 ± 1.7 | 9.5 ± 4.5 | 1.89 | |  |  |
| 6-Phosphogluconate | 3.1 ± 1.2 | 5.8 ± 2.4 | 1.88* | |  |  |
| Citrate | 211 ± 44 | 361 ± 108 | 1.71* | |  |  |
| Spermidine | 2.4 ± 1.3 | 4.0 ± 1.8 | 1.68 | |  |  |
| Lysine | 106 ± 43 | 170 ± 164 | 1.60 | |  |  |
| Creatine | 11,156 ± 1734 | 17,311 ± 2,573 | 1.55*** | |  |  |
| Glutathione (GSH) | 378 ± 104 | 571 ± 111 | 1.51* | |  |  |
| Tryptophan | 25 ± 5.0 | 30 ± 6.0 | 1.23 | |  |  |
| Arginine | 89 ± 22 | 108 ± 16 | 1.21 | |  |  |
| Pyruvate | 87 ± 8.5 | 103 ± 22 | 1.19 | |  | |
| Leucine | 95 ± 10 | 110 ± 18 | 1.16 | |  | |
| Tyrosine | 74 ± 9.2 | 84 ± 13 | 1.13 | |  | |
| Uridine diphosphate-glucose | 12.3 ±1.5 | 14.0 ± 1.7 | 1.13 | |  | |
| Isoleucine | 63 ± 11 | 71 ± 14 | 1.12 | |  | |
| Putrescine | 5.3 ± 1.3 | 5.9 ± 1.7 | 1.11 | |  | |
| Argininosuccinic acid | 2.5 ± 1.4 | 2.7 ± 1.1 | 1.09 | |  | |
| Valine | 194 ± 27 | 210 ± 25 | 1.09 | |  | |
| Threonine | 168 ± 25 | 182 ± 39 | 1.08 | |  | |
| Phenylalanine | 67 ± 5.8 | 72 ± 5.4 | 1.08 | |  | |
| Methionine | 37 ± 3.7 | 39 ± 5.5 | 1.06 | |  | |
| Creatinine | 236 ± 22 | 245 ± 44 | 1.04 | |  | |
| Dihydroxyacetone phosphate | 210 ± 58 | 218 ± 82 | 1.04 | |  | |
| NADH | 9.4 ±0.7 | 9.6 ±0.4 | 1.03 | |  | |
| Spermine | 34 ± 6.2 | 34 ± 4.2 | 1.02 | |  | |
| S-Adenosylmethionine | 6.6 ± 1.1 | 6.7 ± 0.8 | 1.02 | |  | |
| Histidine | 89 ± 20 | 90 ± 18 | 1.02 | |  | |
| Urea | 4155 ± 345 | 4,124 ± 427 | 0.99 | |  | |
| Glycine | 733 ± 102 | 726 ± 70 | 0.99 | |  | |
| Carnosine | 20,891 ± 1114 | 20,534 ± 1,637 | 0.98 | |  | |
| NAD+ | 404 ± 49 | 395 ±38 | 0.98 | |  | |
| Asparagine | 58 ± 18 | 57 ± 10 | 0.98 | |  | |
| Ornithine | 26 ± 8.9 | 25 ± 11 | 0.97 | |  | |
| Serine | 377 ± 85 | 360 ± 81 | 0.96 | |  | |
| N-Acetylglutamic acid | 3.9 ± 0.6 | 3.6 ± 0.5 | 0.92 | |  | |
| Citrulline | 93 ± 25 | 86 ± 14 | 0.92 | |  | |
| NADPH | 9.0 ± 0.9 | 8.2 ± 0.9 | 0.92 | |  | |
| 2-Hydroxyglutaric acid | 8.9 ± 2.4 | 8.1 ± 0.5 | 0.91 | |  | |
| 2,3-Diphosphoglyceric acid | 22 ± 11 | 20 ± 10 | 0.90 | |  | |
| NADP+ | 21.5 ± 5.0 | 19.4 ± 4.8 | 0.90 | |  | |
| β-Alanine | 253 ± 65 | 221 ± 54 | 0.87 | |  | |
| γ-Aminobutyric acid | 9.9 ± 2.2 | 8.4 ± 1.6 | 0.84 | |  | |
| Adenosine triphosphate | 5762 ± 525 | 4,844 ± 798 | 0.84* | |  | |
| Betaine | 227 ± 58 | 190 ± 33 | 0.84 | |  | |
| GDP | 4.7 ± 0.5 | 3.9 ± 0.5 | 0.83* | |  | |
| Glutamine | 1337 ± 297 | 1,102 ± 229 | 0.82 | |  | |
| Aspartate | 187 ± 79 | 154 ± 45 | 0.82 | |  | |
| ADP | 134 ± 13 | 110 ± 35 | 0.82 | |  | |
| Ribulose 5-phosphate | 8.6 ± 2.0 | 6.9 ± 2.6 | 0.81 | |  | |
| Guanosine triphosphate | 98 ± 16 | 79 ± 7.9 | 0.81* | |  | |
| CoA | 13.1 ± 3.3 | 9.9 ± 2.8 | 0.76 | |  | |
| Fructose 1,6-diphosphate | 322 ± 157 | 234 ± 113 | 0.73 | |  | |
| ADP-ribose | 44.4 ±7.6 | 24.5 ± 9.2 | 0.55** | |  | |
| Phosphocreatine | 2952 ± 198 | 1,606 ± 555 | 0.54** | |  | |
| Glutathione disulfide | 251 ± 45 | 127 ± 39 | 0.50*** | |  | |
| 2-Phosphoglyceric acid | 8.5 ± 3.2 | 3.5 ± 1.5 | 0.42* | |  | |
| 3-Phosphoglyceric acid | 101 ± 39 | 39 ± 15 | 0.39* | |  | |
| Glutamate | 2,488 ± 391 | 713 ± 229 | 0.29*** | |  | |
| Phosphoenolpyruvate | 24 ± 13 | 6.2 ± 3.9 | 0.26* | |  | |
| Carnitine | 5,881 ± 899 | 1,474 ± 250 | 0.25*** | | | |

Asterisks show significant differences between before and after exercise by Welch’s t-test (*<0.05, **<0.01, ***<0.001).
